# Retrospective Cohort Study of Frequency and Patterns of Orbital Injuries on Whole-Body CT with Maxillofacial Multi-Slice CT

**DOI:** 10.3390/tomography7030033

**Published:** 2021-08-17

**Authors:** Leonie Goelz, Annika Syperek, Stephanie Heske, Sven Mutze, Norbert Hosten, Michael Kirsch

**Affiliations:** 1 Department of Radiology and Neuroradiology, BG Klinikum Unfallkrankenhaus Berlin, Warener Str. 7, 12683 Berlin, Germany; sven.mutze@ukb.de; 2 Institute for Diagnostic Radiology and Neuroradiology, University Medicine Greifswald, 17475 Greifswald, Germany; Annika.Syperek@med.uni-greifswald.de (A.S.); stephanie.heske@immanuelalbertinen.de (S.H.); norbert.hosten@med.uni-greifswald.de (N.H.); Michael.Kirsch@med.uni-greifswald.de (M.K.)

**Keywords:** orbital trauma, polytrauma, orbital soft tissue injury, maxillofacial, emergency imaging

## Abstract

*Background*: High-impact trauma frequently leads to injuries of the orbit, but literature focusing on the viscerocranium rather than the neurocranium is underrepresented. *Methods*: Retrospective cohort study (2006–2014) at an urban level 1 trauma center assessing the frequency and typical patterns of orbital injuries on whole-body computed tomography (WBCT) with maxillofacial multi-slice CT (MSCT) after severe trauma. (1) Screening of consecutive WBCT cases for dedicated maxillofacial MSCT. (2) Examination by two independent experts’ radiologists for (peri-/)orbital injuries. (3) Case review for trauma mechanisms. *Results*: 1061 WBCT were included revealing 250 (23.6%) patients with orbital injuries. Less than one-quarter (23.3%) of patients showed osseous and 9.5% showed soft tissue injuries. Combined osseous and soft tissue lesions were present in 39.2% of orbital injuries, isolated soft tissue injuries were rare. Single- or two-wall fractures of the orbit were prevalent, and the orbital floor was affected in 67% of fractures. Dislocated extraocular muscles (44.6%), deformation of the ocular globe (23.8%), and elongation of the optic nerve (12.9%) were the most frequently soft tissue findings. Vascular trauma was suspected in 15.8% of patients. *Conclusions*: Orbital trauma was confirmed in 23.6% of cases with suspected facial injuries after severe trauma. Concomitant soft tissue injuries should be excluded explicitly in cases with orbital fractures to prevent loss of vision or ocular motility.

## 1. Introduction

Orbital injuries pose a serious threat to a patient’s vision and ocular motility. Existent literature describes typical osseous and soft tissue injuries encountered after orbital trauma [1,2], but only a few studies have reported on orbital injuries of the entity of severely injured patients [3,4]. Head trauma is one of the most observed injuries in polytrauma patients [5]. Studies usually focus on the injuries of the neurocranium due to their imminent risk to the patients’ lives. Nonetheless, the severity of traumatic brain injuries is known to correlate with the presence and severity of concomitant facial trauma [6,7]. Extensive periorbital swelling and reduced vigilance of the polytraumatized patients can inhibit accurate ophthalmological testing. In these cases, radiological imaging becomes essential for the quick and three-dimensional evaluation of the orbital cavity. Osseous orbital injuries can best be identified on high-resolution multi-slice CT (MSCT) with bone windowing [8]. Additional multiplanar reconstructions of the coronal and sagittal planes enable detailed assessment and facilitate diagnostics of some injuries [9]. Even though MRI provides superior soft tissue contrast when compared to MSCT, and ultrasonography can survey the ocular globe precisely, MRI is less relevant in the acute setting due to long acquisition times as well as the possible risk of dislocation of metallic foreign bodies [10], and ultrasonography is contraindicated in cases of suspected globe rupture [11]. Nevertheless, contrast-enhanced MSCT can depict most soft tissue injuries accurately [12,13], and even hint to underlying carotid-cavernous fistulas (CCF) when dilated superior ophthalmic veins, exophthalmos, and orbital edema are present [14]. Furthermore, radiopaque intraorbital foreign bodies including glass fragments can be identified with a sensitivity of up to 90% on MSCT [2,15]. Wooden foreign bodies can be harder to differentiate but they may present as streaky hypodensities, a correlate of air bubbles in the wood [16].

The German S3 guideline for the treatment of polytrauma and the severely injured was first published in 2011 in an interdisciplinary effort of 20 different medical societies involved in the care of severely injured patients and revised in 2016 [17]. The guideline contains clear indications each leading independently to immediate initiation of whole-body CT (WBCT), namely, pathological vital signs, at least two relevantly injured body regions, and/or relevant trauma mechanisms such as severe road traffic accidents and falls from heights of more than 3 m [18]. Imaging studies with comparable patient cohorts after severe trauma focusing on orbital injuries have not been published in recent years. However, discussion of prevalence and incidence in real-life study populations is essential to sensitize radiologist and clinicians for orbital injuries in this patient cohort. A deepened insight into the specifics of these injuries after high-impact trauma may help to further standardize trauma protocols and guidelines in the future.

Therefore, this current study seeks to assess the frequency, typical patterns of osseous and soft tissue injuries of the orbit on WBCT with maxillofacial MSCT after severe trauma, and to evaluate trauma mechanisms in this patient category of a level 1 trauma center.

## 2. Materials and Methods

### 2.1. Study Design and Population

This cohort study was approved by the Ethics committee of University Medicine Greifswald (registration number: BB 115/20) and conducted in accordance with the Declaration of Helsinki of 2013. Informed consent was waived due to the retrospective design of the study.

**Figure 1:**
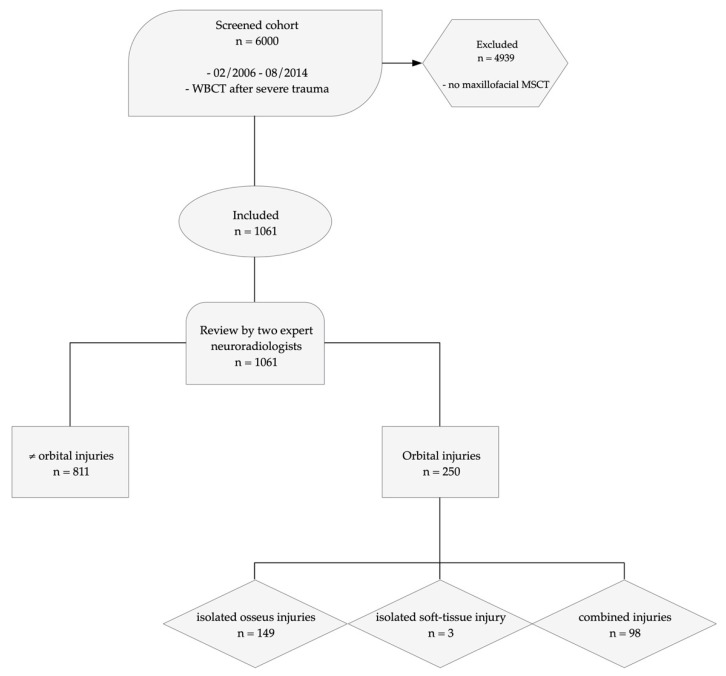
Study protocol visualizing the workflow according to the recommendations of the STROBE—Strengthening the Reporting of Observational Studies in Epidemiology Initiative [19] showing patient screening, inclusion, exclusion, and the results of analysis by two neuroradiologists (WBCT = whole-body CT, MSCT = multi-slice CT).

From February 2006 to August of 2014, 6000 WBCT were performed at a large metropolitan trauma center due to suspected polytrauma according to the German S3 guideline for the treatment of polytrauma and the severely injured and screened for additional maxillofacial MSCT [17]. One-thousand-and-sixty-one cases were identified and included in the study (Figure 1).

### 2.2. CT Protocol and Image Analysis

The patients received a non-contrast head CT, a contrast enhanced WBCT with arms crossed in front of the abdomen, and an additional maxillofacial MSCT due to suspected facial trauma. Axial images (0.75 mm) were acquired following a standardized polytrauma protocol comprised of arterial scans from the orbital roof to the aortic arch, and another scan from chest to pelvis following a split-bolus protocol [20]. Images were acquired on three different CT scanners (all: Philips, Eindhoven, The Netherlands): 645 were performed on a 64-slice scanner (Diamond Select Brilliance), 395 on a 128-slice scanner (Ingenuity Elite), and 21 on a 16-slice scanner (Diamond Select Brilliance). Multiplanar reconstructions of axial, coronal, and sagittal planes (slice thickness 2 mm) were created and stored with the raw data in the Picture Archiving and Communications System (PACS) iSite^®^ Radiology (Philips, Eindhoven, The Netherlands).

Two neuroradiologists with more than twenty years of work experience each (S.M., M.K.) reviewed the images independently. In cases of disagreement, consensus was reached via discussion.

### 2.3. Diagnostic Criteria

During image analysis, the presence of the following criteria was assessed: osseous lesions of the orbit including the optic canal, soft tissue lesions, post-traumatic hemorrhage or edema, and intraorbital foreign bodies.

In detail, the number of affected orbital walls and location of fractures was examined and soft tissue injuries were further partitioned into four sub-categories: (1) injuries of the ocular globe and lens (deformation or rupture of the ocular globe, dislocation of the lens, intraocular foreign bodies), (2) injuries of the extraocular muscles (dislocation or displacement, piercing injuries by bony fragments, intramuscular foreign bodies), (3) injuries of the optic nerve (elongation, edema, otherwise altered morphology, damage inflicted by foreign bodies), and (4) trauma associated abnormalities of the orbital vessels (direct CCF, engorged superior ophthalmic veins).

### 2.4. Statistical Analysis

CT findings and medical records were documented using an Excel worksheet (Excel 2010; version 14, Microsoft Cooperation, Redmond, WA, USA). The statistical analysis system (SAS^®^ 9.4 for Windows 10 [SAS Institute Inc., Cary, NC, USA]) was employed for all statistical analysis by a biostatistician. Descriptive statistics for continuous variables were expressed as arithmetic mean, median, standard deviation (SD), minimum and maximum (range), absolute (*n*) and relative (%) proportions. Categorical variables were expressed as absolute (*n*) and relative (%) proportions.

Missing values were not imputed but presented for each variable if existing. Our reporting adhered to the Strengthening the Reporting of Observational Studies in Epidemiology (STROBE-) Initiative (Figure 1) [19].

## 3. Results

### 3.1. Study Population

Seventy-six percent of the included 1061 patients were males (*n* = 806) with a mean age of 44.5 ± 7.3 years (range: 3 to 98 years). The most common mechanism of injury was road traffic accidents (54.7%). Falls from heights > 3 m (28.8%) and assaults (3.8%) occurred less often. In 26.4% of road traffic accidents patients were inside a car, 10.4% were bicyclists, 9.0% were pedestrians, and 8.9% were motorcyclists (Table 1).

### 3.2. Frequency of Orbital Injuries

Two-hundred-and-fifty patients (23.56%) with orbital injuries were identified: 149 (14.0%) suffered from isolated orbital fractures, 3 (0.3%) showed isolated soft tissue injuries, and in 98 cases (9.2%) combined injuries were present. Table 2 illustrates the distribution of osseus and soft tissue injuries in the study population (Table 2).

#### 3.2.1. Osseous Injuries

Two-hundred-and-forty-seven patients suffered fractures of the orbit. They occurred in an even distribution on both sides and bilateral fractures were diagnosed in 78 (31.6%) of these patients.

Single-wall fractures of the orbit were the most common lesion with 91 cases (36.8%). Injuries involving multiple orbital walls became increasingly rare the more orbital walls were affected, identifying only eight cases (3.2%) with four wall fractures (Table 3).

The orbital floor was affected in 165 patients with orbital fractures (66.8%), the orbital roof in 116 patients (47.0%), the lateral wall in 106 patients (42.9%), and the medial wall in 78 patients (31.6%) of patients showing fractures in this region. Bilateral fractures affected a single wall of each orbit in 12 patients, while the remaining 66 showed a multitude of complex fracture patterns involving two or more orbital walls.

#### 3.2.2. Soft Tissue Injuries

One-hundred-and-one patients suffered one or more soft tissue injuries of the orbit. Extraocular muscle injuries were described commonly in 54 (53.5%) of these cases, injuries of the globe and lens in 38 cases (37.6%), and injuries of the optic nerve in 24 cases (23.8%). Orbital vessel damage was suspected in 16 patients with soft tissue injuries (15.8%). Table 4 illustrates the frequencies of soft tissue injuries in the four main categories.

**Figure 2:**
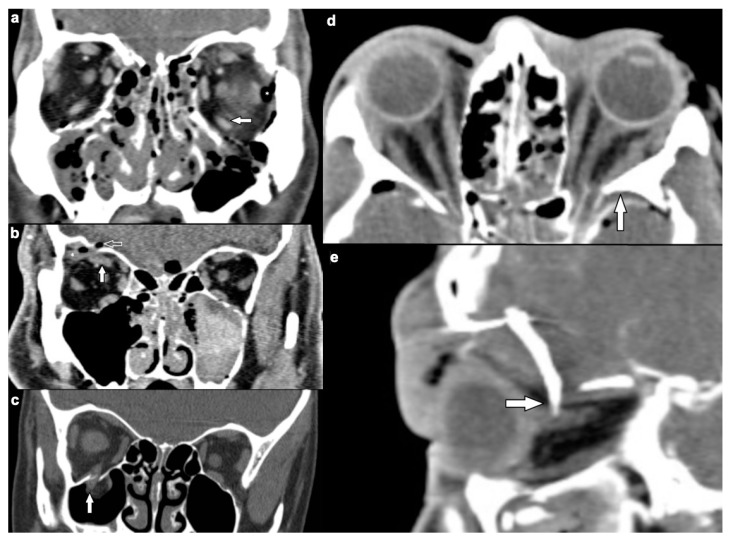
(**a**–**c**) Dislocated extraocular muscles. CE-MSCT, soft tissue window, coronal reformations. (**a**) Dislocation of the left inferior rectus muscle (white solid arrow) due to orbital floor fracture. Asterisk indicates orbital emphysema. (**b**) Displacement of the right superior rectus muscle (solid white arrow) with orbital roof fracture (white outlined arrow) and intraorbital hemorrhage (asterisk). Concomitant left hematosinus maxillaris due to midface fractures. (**c**) Right inferior rectus muscle (white solid arrow) incarcerated in a so-called “trapdoor fracture” of the orbital floor. (**d**,**e**) Extraocular muscles pierced by bone fragments. CE-MSCT, soft tissue window. (**a**) Axial reformation. Left lateral rectus muscle pierced by a fragment of the greater sphenoid wing (white arrow). (**b**) Sagittal reformation. Right superior rectus muscle pierced by a frontal bone fragment (white arrow), which also displaces the optic nerve (CE-MSCT = contrast-enhanced multi-slice CT).

A dislocation of orbital muscles occurred in 45 of patients with soft tissue injury (44.6%) (Figure 2a–c). In eight (7.9%) patients, extraocular muscles had been pierced by bony fragments (Figure 2d,e).

In a single case, muscle trauma was inflicted by a foreign body (1.0%).

**Figure 3:**
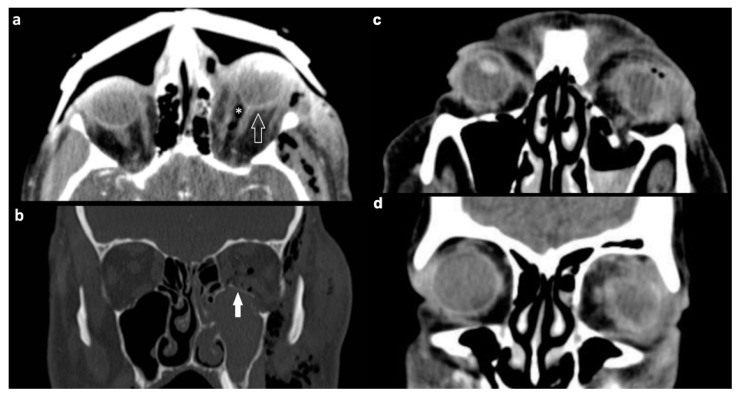
(**a**,**b**) Orbital compartment syndrome after blow-in fracture of the left orbital floor. NE-MSCT (**a**) Soft tissue window, axial reformation. Mass effect of a dislocated orbital floor fragment leading to left-sided exophthalmos with elongation of the optic nerve and deformation of the ocular bulb (globe tenting, indicated by hollow arrow). The posterior globe angle is reduced to 117° in the left globe, compared to 139° on the right side. Asterisk indicates intraconal air bubbles. (**b**) Bone kernel, coronal reformation. Solid arrow indicates the orbital floor fragment which is dislocated upwards into the orbital cavity. (**c**,**d**) Complete rupture of the left globe with irregular shape, volume loss, and air bubbles in the anterior chamber. NE-MSCT, soft tissue window in (**a**) Axial and (**b**) coronal reformation (NE-MSCT = non-contrast-enhanced multi-slice CT).

Deformation of the ocular globe was described in 24 (23.8%) patients (Figure 3a) while a partial or complete rupture of the globe was seen in only seven (6.9%) patients with soft tissue injury (Figure 3c,d). Four out of seven cases of rupture occurred in conjunction with fractures of all four orbital walls (Figure 3b).

**Figure 4:**
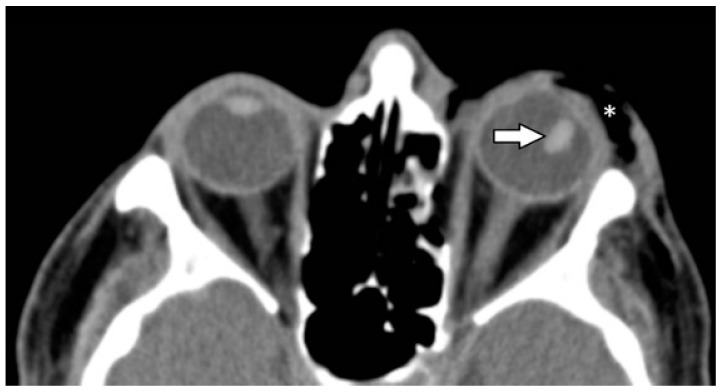
Dislocated left lens (indicated by arrow) and lateral rotation of about 70 degrees. Asterisk (*) indicates periorbital soft tissue emphysema due to a concomitant orbital floor fracture. NE-MSCT, soft tissue window, axial reformation (NE-MSCT = non-contrast-enhanced multi-slice CT).

Six patients (6.9%) showed a dislocated lens (Figure 4).

**Figure 5:**
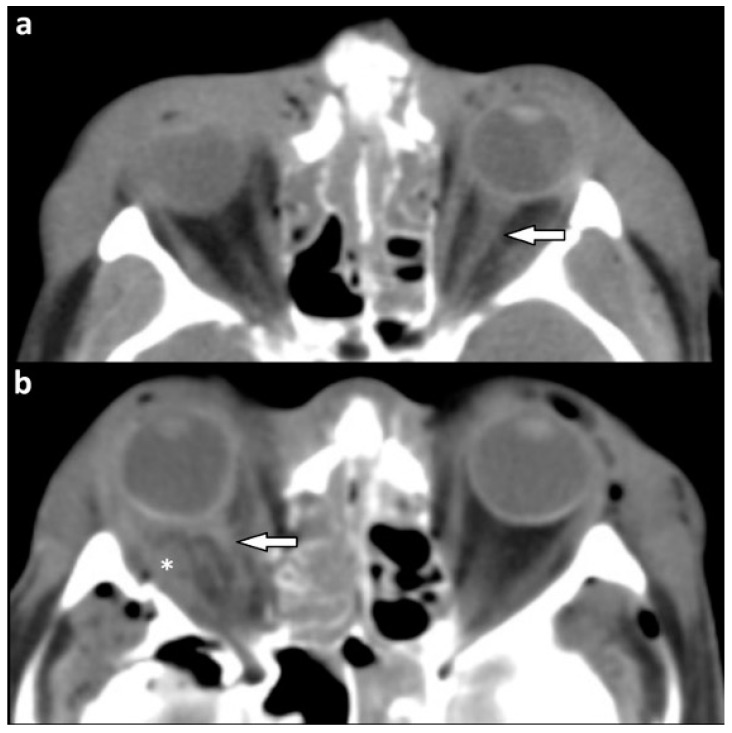
Elongated optic nerves associated with traction trauma. NE-MSCT, soft tissue window, axial plane. (**a**) The left optic nerve (white arrow) appears elongated and hyperdense due to intraneural bleeding. (**b**) Swelling and deformation of the right optic nerve (white arrow) and retrobulbar hematoma (asterisk) in a complex midface fracture involving all orbital walls (NE-MSCT = non-contrast-enhanced multi-slice CT).

Thirteen patients (12.9%) were diagnosed with an elongation of the optic nerve compared to the contralateral side, and in 10 cases (9.9%), its shape was otherwise altered (Figure 5).

The optic nerve was punctured by a foreign body in one patient (1.0%).

**Figure 6:**
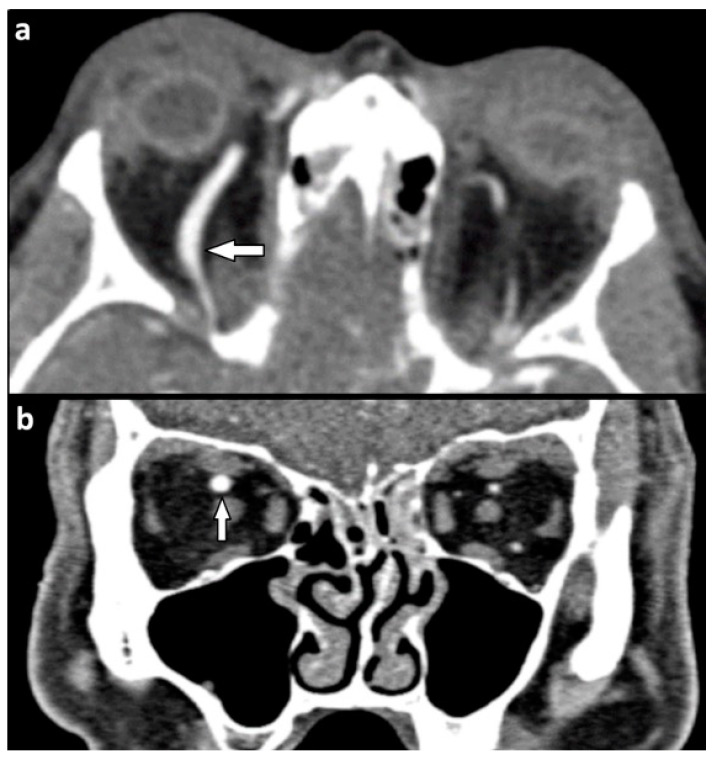
Prominent right ophthalmic vein (white arrows). Compared to the contralateral vein, the vessel appears engorged. CE-MSCT, soft tissue window. (**a**) Axial reformation. (**b**) Engorged superior ophthalmic vein and exophthalmos of the right globe hinting at post-traumatic CCF (CE-MSCT = contrast-enhanced multi-slice CT, CCF = carotis-cavernous fistula).

The superior ophthalmic vein was enlarged in 10 (9.9%) patients with soft tissue injuries (Figure 6) as an indirect sign hinting at CCF.

A direct CCF was identified in six (5.9%) individuals.

**Figure 7:**
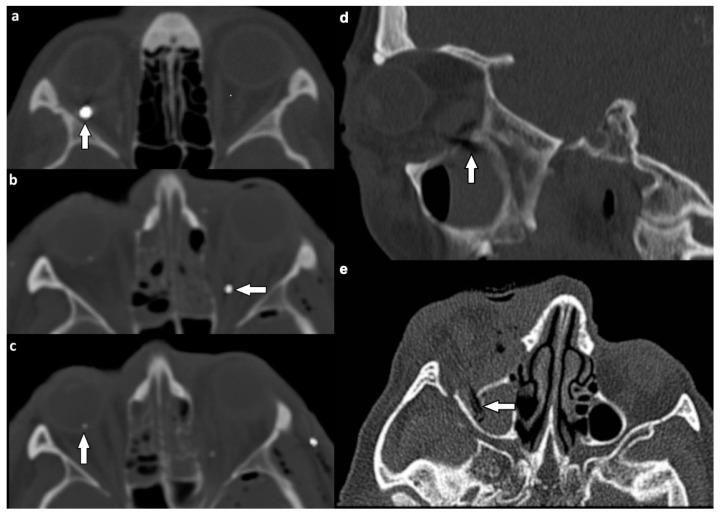
Intraorbital foreign bodies. NE-MSCT, bone window, axial reformation. (**a**) Metallic foreign body (white arrow) within the right lateral rectus muscle. (**b**,**c**) Multiple metallic foreign bodies in one patient (**b**) near the left optic nerve (white arrow) and (**c**) within the rear wall of the ocular globe (white arrow). (**d**,**e**) Wooden foreign body in the right orbit (white arrows) piercing through the orbital floor into the right maxillary sinus. Concomitant hematosinus and pronounced proptosis due to retrobulbar hematoma and swelling. Hypodense air bubbles entrapped within the otherwise undetectable dry wood. NE-MSCT, bone window and bone kernel in (**d**) sagittal and, (**e**) axial reformation (NE-MSCT = non-contrast-enhanced multi-slice CT).

Intraorbital foreign bodies were identified in extraocular muscles, the optic nerve, and the ocular globe in three individuals (3%) (Figure 7).

## 4. Discussion

The frequency of orbital injuries on WBCT with maxillofacial MSCT after suspected facial trauma was 23.6% in this cohort of patients after severe trauma. Among all screened polytrauma patients, orbital injuries were diagnosed in 4.2% cases. This figure is lower than a previously reported prevalence of 7.5% for orbital injuries among polytrauma patients [3]. However, the methods of a CT imaging study differ significantly from a retrospective review of patient records. Another reason for a lower proportion of orbital injuries in the current patient collective might be differences in study design. The selection of the screening cohort of the current study was based on the accordance with the German S3 guideline for the treatment of polytrauma and the severely injured [17] and consisted of a typical, real-life cohort of all trauma patients who were examined via WBCT during the study period. An approach through patient records with inclusion of patients strictly depending on the severity of injuries increases the pretest probability of the examination results. Nevertheless, most patients included in this study (83.5%) suffered severe accidents through road traffic or significant falls, therefore the reported prevalence mirrors the potential for orbital injuries after high-impact accidents. In contrast, by adding dedicated maxillofacial MSCT to the inclusion criteria, the prevalence increased to 23.6% as these scans are not routinely included in the polytrauma protocol but only in cases of suspected facial trauma.

In the current patient collective, 23.3% of included patients suffered osseous orbital injuries. Most of these fractures (66.8%) involved the orbital floor and most patients (63.2%) showed more than one orbital wall fracture. These findings concur with prior reports about patients with isolated head and orbital trauma [20,21,22] and underline the importance of carefully identifying each fracture on CT imaging to determine the appropriate therapeutic regime [23,24]. Concomitant soft tissue pathologies such as the prolapse of orbital fat into the maxillary sinus or the entrapment of extraocular muscles are recurrent findings in these cases [25]. The orbital roof showed the second most fractures in this analysis, and even though it is the thinnest orbital wall, the medial wall was least likely to be fractured. Reyes et al. described the medial wall as just as fragile as the orbital floor, but their patients were injured during physical aggression in most cases and rarely during traffic accidents. It appears conclusive that the vector of force applied to the orbit during trauma is relevant for fracture patterns. Interestingly, facial trauma in children led to orbital roof fractures most often, followed by lateral and medial wall fractures [26]. Elasticity of the bone and development of facial structures, and thus age distribution in a patient cohort seems relevant for the distribution of orbital wall fracture as well.

Nearly one-tenth (9.5%) of cases of soft tissue injuries were described by the reviewers. Almost all soft tissue injuries were accompanied by various orbital fractures, while isolated soft tissue lesions occurred in only three (0.3%) cases. Even though orbital soft tissue injuries are less common than orbital fractures, they require a swift and precise diagnosis due to their potentially severe complications and long-term consequences [25]. With a 44.6% prevalence among soft tissue injuries, dislocated extraocular muscles were the most common findings on MSCT. The high frequency of occurrence of these injuries is well explained by the simultaneous existence of orbital fractures and the risk of dislocation of extraocular muscles by fracture fragments [13]. These injuries can prompt reduced ocular motility and permanent visual limitations [8]. The second most common soft tissue injury was a deformation of the globe in 23.8% of patients with soft tissue lesions. The most severe form of ocular bulb lesion, a partial or complete rupture of the globe, was seen in 6.9% of cases. According to prior studies, the sensitivity of CT imaging regarding the diagnosis of globe rupture is 67–76% [9]. In consequence, surgical exploration remains an essential method for exclusion of globe rupture if suspicious CT findings such as intraocular hemorrhage, lens dislocation, intraocular foreign bodies, intraocular gas, globe deformity, and wall irregularity are present [27].

One of the major challenges in orbital trauma imaging is identifying injuries that may lead to orbital compartment syndrome (OCS), a condition characterized by elevated intraorbital pressure which can lead to permanent loss of vision in as little as 100 min via retinal or optic nerve ischemia [28]. Imaging signs hinting to elevated intraorbital pressure are proptosis, the elongation of the optic nerve, and a characteristic deformation of the globe referred to as globe tenting. In globe tenting, the posterior aspect of the globe loses its rounded outline and is deformed into a conical shape, resembling a tent, with the posterior globe angle decreasing to below 130 degrees (Figure 3a) [29]. It has been suggested that posterior globe angles below 120 degrees correlate with negative outcomes and constitute an indication for orbital decompression [30].

Intraorbital foreign bodies were described in only three patients of orbital injuries. Dedicated literature reports much higher frequencies of these injuries of up to 17% in cases of orbital trauma [10]. Therefore, a general understanding of the different types of foreign bodies, the difficulty to detect wooden objects (Figure 7d,e), and their appearance on CT imaging is necessary. If radiopaque objects are excluded and CT scans are non-conclusive despite the presence of clinical symptoms, MRI should be employed as it provides better soft tissue contrast resolution [4].

Finally, CCF was suspected in 10 patients (9.9%) of patients with soft tissue injuries because of a dilatation of the superior ophthalmic vein. With an incidence of only 0.2%, CCF are very rare vasculopathies after trauma [31]. Nevertheless, their typical findings, a dilatation of the superior ophthalmic vein, as well as proptosis, enlarged cavernous sinus and extraocular muscles, and orbital edema should initiate further invasive diagnostics through cerebral angiography [12,14].

**Figure 8:**
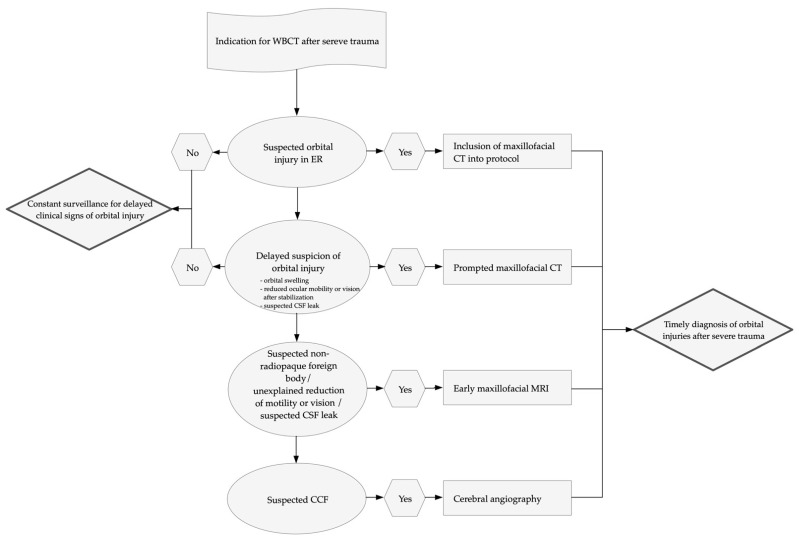
Exemplary protocol for diagnosis and treatment of orbital injuries. The figure highlights the importance of thorough initial clinical and radiological examinations and of an ongoing surveillance for delayed clinical sings of orbital injuries to allow for prompt or early imaging via CT, MRI, or cerebral angiography in the acute and subacute phase after severe trauma (WBCT = whole-body CT, CSF = cerebrospinal fluid, CCF = carotid-cavernous fistula).

In summary, the likelihood of suffering orbital injuries and the highly disabling consequences of missed injuries demands for specific clinical examinations and a strict surveillance of patients after severe trauma to initiate immediate maxillofacial CT. Delayed or unexplained clinical signs, suspected non-radiopaque foreign objects, suspected cerebrospinal fluid (CSF) leaks, or CCF should prompt imaging (CT, MRI, or cerebral angiography) in the subacute phase after polytrauma. Ongoing surveillance for orbital injuries in the intensive care unit could be beneficial in reducing the relevant number of missed orbital injuries [32]. Developing and evaluating protocols for diagnosis and treatment of orbital injuries after severe trauma and amending trauma guidelines could support clinicians and radiologists routinely in the future (Figure 8).

Certain limitations of this study must be addressed. First, the retrospective study design is susceptible to selection bias. By adhering to the STROBE standards [19] and complying with the German S3 guideline for the treatment of polytrauma and the severely injured [17], transparency of the inclusion process of consecutive patients and a clear definition of the patient cohort at a level 1 trauma center act as countermeasures. Second, the exploratory fashion of this study promotes descriptive results which are harder to compare to existing literature with differing patient populations. At last, the design of the study allowed for an inclusion of a large patient cohort, but a concrete prevalence for all 6000 polytrauma patients cannot be provided and the given figure merely serves as an estimate among these patients.

## 5. Conclusions

Orbital injuries occur frequently in real-life after suspected polytrauma and should therefore be anticipated by clinicians and radiologists. Periorbital swelling and reduced vigilance of patients often inhibit accurate ophthalmologic testing. Thus, including maxillofacial MSCT into the WBCT protocol plays a crucial role in the diagnostic workup of orbital trauma. Concomitant soft tissue injuries should be excluded explicitly in cases with orbital fractures to prevent loss of vision or ocular motility due to delayed treatment.

## Figures and Tables

**Table 1:** Distribution of sex, age, and trauma mechanisms in the study population.

	Characteristic	*n* (%)
**Sex**	male	806 (76)
	female	255 (24)
**Age in Years**	0–9	6 (0.6)
	10–19	93 (8.8)
	20–29	219 (20.6)
	30–39	140 (13.2)
	40–49	174 (16.4)
	50–59	187 (17.6)
	60–69	93 (8.8)
	70–79	85 (8.0)
	80–89	59 (5.6)
	90–99	5 (0.5)
**Trauma Mechanism**	Road traffic accidents	581 (54.7)
	- car (driver/passenger)	280 (26.4)
	- bicyclist	110 (10.4)
	- motorcyclist	95 (8.9)
	- pedestrian	96 (9.0)
	Fall from heights > 3 m	305 (28.8)
	Assault	40 (3.8)
	Miscellaneous	135 (12.7)

**Table 2:** Relative frequency of osseous and soft tissue injuries of the orbit among 250 patients with confirmed orbital trauma and among all included patients with maxillofacial MSCT.

		*n*	Included Patients (*n* = 1061)	Confirmed Orbital Trauma (*n* = 250)
			%	%
**All orbital injuries**	total	250	23.6	100.0
**Osseous injuries**				
	total	247	23.3	98.8
	isolated	149	14.0	59.6
**Soft tissue injuries**				
	total	101	9.5	40.4
	isolated	3	0.3	1.2
**Combined injuries**	total	98	9.2	39.2

**Table 3:** The distribution of fractures showed no significant difference between the left and right orbit.

Type of Fracture	Right Orbit *n* (%)	Left Orbit *n* (%)	Total *n* (%)
single wall	51 (58.0)	40 (49.4)	91 (36.8)
two walls	21 (23.9)	22 (27.2)	43 (17.4)
three walls	13 (14.8)	12 (14.8)	25 (10.1)
four walls	2 (2.2)	6 (7.4)	8 (3.2)
four walls and orbital apex	1 (1.1)	1 (1.2)	2 (0.8)
bilateral fractures			78 (31.6)
total	88 (35.6)	81 (32.8)	247 (100)

**Table 4:** Types and frequency of orbital soft tissue injuries showed a dominance of extraocular muscle injuries.

Location of Soft Tissue Injury	Type of Lesion	*n* (%)
**Extraocular muscles**		**54 (53.5)**
	dislocation	45 (44.6)
	pierced by bone fragment	8 (7.9)
	intramuscular foreign body	1 (1.0)
**Ocular globe and lens**		**38 (37.6)**
	Deformed globe or vitreous body	24 (23.8)
	rupture of ocular globe	7 (6.9)
	dislocated lens	6 (5.9)
	intraconal foreign body	1 (1.0)
**Optic nerve**		**24 (23.8)**
	elongation	13 (12.9)
	otherwise altered morphology	10 (9.9)
	pierced by foreign body	1 (1.0)
**Orbital vessels**		**16 (15.8)**
	dilated superior ophthalmic vein	10 (9.9)
	direct carotid cavernous fistula	6 (5.9)

## Data Availability

Data are contained within the article. Raw data are available on request due to privacy restrictions.
